# Asymptomatic bacteriuria in older adults: the most fragile women are prone to long-term colonization

**DOI:** 10.1186/s12877-019-1181-4

**Published:** 2019-06-21

**Authors:** Michael Biggel, Stefan Heytens, Katrien Latour, Robin Bruyndonckx, Herman Goossens, Pieter Moons

**Affiliations:** 1 Laboratory of Medical Microbiology, University of Antwerp, Universiteitsplein 1, building S, 2610 Wilrijk, Antwerp, Belgium; 2 Department of Family Medicine and Primary Health Care, Ghent University, Ghent, Belgium; 3 Operational Directorate Epidemiology & Public Health, Sciensano, Brussels, Belgium; 4 Department of Public Health and Primary Care, KU Leuven, Leuven, Belgium; 5 Interuniversity Institute for Biostatistics and Statistical Bioinformatics (I-BIOSTAT), Hasselt University, Hasselt, Belgium

**Keywords:** Asymptomatic bacteriuria, Urinary tract infection, Older adults

## Abstract

**Background:**

The diagnosis of urinary tract infections (UTIs) in institutionalized older adults is often based on vague symptoms and a positive culture. The high prevalence of asymptomatic bacteriuria (ABU), which cannot be easily discriminated from an acute infection in this population, is frequently neglected, leading to a vast over-prescription of antibiotics. This study aimed to identify subpopulations predisposed to transient or long-term ABU.

**Methods:**

Residents in a long-term care facility were screened for ABU. Mid-stream urine samples were collected during two sampling rounds, separated by 10 weeks, each consisting of an initial and a confirmative follow-up sample.

**Results:**

ABU occurred in approximately 40% of the participants and was mostly caused by *Escherichia coli*. Long-term ABU (> 3 months) was found in 30% of the subjects. The frailest women with urinary incontinence and dementia had drastically increased rates of ABU and especially long-term ABU. ABU was best predicted by a scale describing the functional independence of older adults.

**Conclusions:**

Institutionalized women with incontinence have ABU prevalence rates of about 80% and are often persistent carriers. Such prevalence rates should be considered in clinical decision making as they devalue the meaning of a positive urine culture as a criterion to diagnose UTIs. Diagnostic strategies are urgently needed to avoid antibiotic overuse and to identify patients at risk to develop upper UTI.

**Electronic supplementary material:**

The online version of this article (10.1186/s12877-019-1181-4) contains supplementary material, which is available to authorized users.

## Background

The diagnosis of urinary tract infections (UTIs) is common in long-term care facilities (LTCFs) and was shown to account for 22% of all infections and more than 50% of the antibiotic prescriptions in this setting [1]. However, in institutionalized older adults, UTI is difficult to differentiate from asymptomatic bacteriuria (ABU). ABU prevalence rates in LTCFs are high, ranging from 25 to 50% in women and from 15 to 40% in men [2]. Such high ABU prevalence rates obviously compromise the value of a positive urine culture as a diagnostic criterion for UTI. In addition, assessing symptoms of a UTI is challenging in this population. Institutionalized older adults, especially those with mental impairment or chronic symptoms, are often incapable of recognizing or communicating the presence of symptoms [3, 4]. Moreover, this geriatric population frequently exhibits atypical manifestations of acute disease and presents with nonspecific symptoms. As UTI can cause severe infections, practitioners are prone to diagnose a UTI relying solely on vague symptoms such as changes in behavior or changes in the appearance of urine rather than typical symptoms of a UTI such as dysuria, frequency or urgency [4, 5]. Residents of LTCFs thus frequently receive antibiotics due to a suspected infection, resulting in a significant overuse of antimicrobials [6]. Treatment of ABU in this population is however not recommended because of potential adverse effects and a lack of efficacy in preventing subsequent UTIs [7, 8]. Moreover, there is increasing evidence that ABU protects against infection [9, 10].

Because of these ambiguities, understanding which residents are prone to develop ABU is important and can help physicians to guide antibiotic treatment. While urine cultures from subjects with a high risk of having ABU should be interpreted with care, urine cultures from low-risk groups have a higher diagnostic value. Older studies identified age, incontinence, functional disability, mental status and mobility as risk factors that are associated with an increased chance of having ABU [11, 12]. Although there are discrepancies between these studies, in general, more frail subjects have a higher risk of ABU [13]. Longitudinal studies in the older adults report that the overall prevalence of ABU is rather static, but with high turnover rates, and conclude that ABU is a rather transient phenomenon with only a minority of people presenting with persistent colonization [11, 14, 15].

In this study, we identified prevalence rates, causal species and risk factors associated with ABU and long-term ABU in a LTCF. In addition, we recorded UTI episodes of all participants during the study period and 6 months following the last sample collection to investigate a possible preventive effect of ABU on UTI occurrences. A policy of the LTCF to avoid the prophylactic use of antibiotics allowed to evaluate the occurrence of asymptomatic bacteriuria with only few participants excluded due to antibiotic interference.

## Methods

### Study population

The study was conducted in a 146-bed LTCF in Destelbergen, Ghent, Belgium, between April and June 2017. The LTCF consists of a nursing home (101 beds), housing residents who are dependent in their activities of daily living in varying degrees, and a residential home (45 beds), accommodating older people with a higher degree of self-reliance. Residents permanently living in the LTCF, > 65 years of age and agreeing to participate were eligible for the study. Participants who were catheterized, had a UTI or received systemic antibiotic treatment during or within the week prior to each urine sampling round were excluded from the analysis.

### Definitions

Urine cultures were considered positive if they showed growth of one or multiple species in concentrations of ≥10^5^ CFU/ml. Regardless of gender, ABU was defined as the presence of at least 10^5^ CFU/mL of the same species isolated from two consecutive samples collected within 10–18 days from a subject without signs or symptoms of a UTI, a definition which is in line with IDSA guidelines [16]. Currently, there is no explicit definition of long-term ABU, and the time frames used in previous studies range from 3 weeks to several months. In this study, long-term ABU was defined as having ABU with the same species in two consecutive sampling rounds separated by 10 weeks, i.e. bacteriuria with the same species in four consecutive samples. Participants who had ABU in only one of the two sampling rounds, i.e. lost or acquired ABU during the study period, were defined as having transient ABU. This evaluation was only applicable to participants who were included in the analysis of both sampling rounds.

### Sample and data collection

**Fig. 1:**
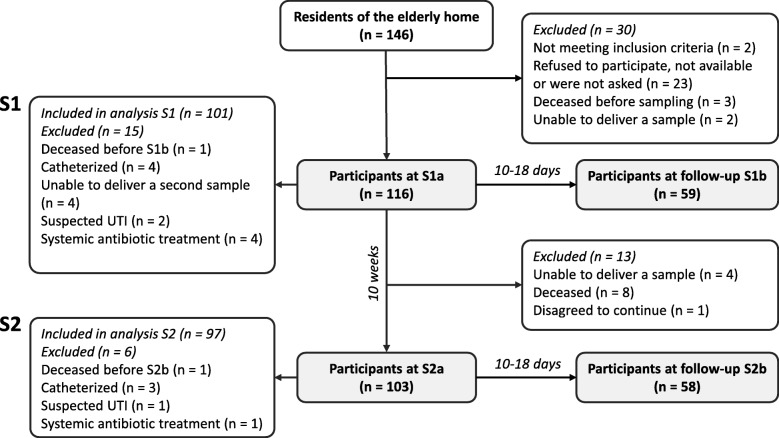
Study flow diagram. Urine samples were collected in two sampling rounds, S1 and S2, each consisting of an initial and a follow-up sample to confirm ABU. Follow-up samples (S1b/S2b) were only requested from participants with possible ABU, i.e. who had a positive urine culture in S1a/S2a

Urine samples were collected in two sampling rounds: S1 and S2. Each sampling round consisted of an initial screening (S1a and S2a) for bacteriuria followed by a second sampling point (S1b and S2b) 10–18 days later to confirm the continued presence of the same species and thus asymptomatic bacteriuria (Fig. 1). Only subjects with bacteriuria in S1a/S2a were asked for the confirmative sample S1b/S2b. Participants who were unable to give the confirmative sample were not included in the analysis of the respective sampling round. The two sampling rounds were separated by 10 weeks and lasted 2–3 weeks, resulting in a total study time of approximately 3 months. For the analysis of long-term and transient ABU, only those participants who were included in the analysis of both S1 and S2 were considered in the evaluation.

Participants and nurses were instructed on the collection of clean-catch mid-stream urine samples. Samples were obtained either through self-collection or by assisting nurses. The urine samples were refrigerated, transported on ice to the microbiology lab and processed within 12 h of collection. In the lab, urine samples were 10-fold serially diluted in sterile saline (0.9% NaCl) and analyzed by standard quantitative urine culture on cystine lactose electrolyte deficient agar (CLED, Becton Dickinson) and Columbia blood agar (OXOID) supplemented with 5% horse blood. Plates were incubated for 18–24 h at 37 °C, and resulting colonies were identified using MALDI-TOF mass spectrometry (Bruker Biotyper).

During the study, several questionnaires had to be completed. At baseline, resident characteristics (age, gender, physical mobility, Belgian Evaluation Scale category) and comorbidities (i.e. dementia, diabetes, urinary incontinence) were collected for each participant. Incontinence was categorized into ‘continuous incontinence’ and ‘occasional incontinence’ for irregular undeliberate loss of urine.

The Belgian Evaluation Scale (BES) is an adaption of the Index of Independence in Activities of Daily Living (ADL) [17] with additional evaluations on orientation in time and space and four instead of three score categories for each function [18]. It classifies older adults in different levels of dependency based on their scores for important activities of daily living and their ability to orient themselves in time and living environment. The 5 categories are O (completely independent, no cognitive impairment) or A, B or C with dependency increasing from A (help needed for washing and/or clothing), over B (additionally help needed for transfers and/or toileting) to C (additionally being incontinent and/or depending on help with feeding), with difficulties in orientation in time and living environment increasing the category by one level. A fifth category, i.e. Cd, stands for level C plus a high degree of disorientation [18]. At each sampling point, information on suspected infections and catheterization (intermittent, indwelling or external) during or in the week prior to urine sampling was documented. At the end of the study, the use of antimicrobials (compound and indication), diuretics and analgesics were recorded covering the entire study period and the 2 weeks prior to the first sample collection.

Suspected UTIs in all participating residents were monitored during the study and over a period of 6 months following the last sample collection. Cases were reported by nurses or the attending physician, and signs or symptoms were documented. The modified McGeer surveillance criteria for UTI [19] were applied by the researchers to confirm the diagnosis.

### Statistical analysis

GraphPad Prism 6 was used for data visualization. Statistical analyses were performed using SPSS 24.0 and SAS 9.4. Age was categorized as > 85 or ≤ 85 years. Comparisons of proportions were tested using the chi-square test and relative risks (RR) were calculated using cross tables. Variables with *p* values < 0.2 were then considered for multivariate analysis. Because samples in sampling rounds S1 and S2 were collected from the same cohort, observations from the same patient were expected to be correlated. To account for this, a generalized estimating equations (GEE) model was constructed using the ABU status as a binary outcome, a logit link function and an independent working correlation. To calculate associations of risk factors with long-term ABU, a multiple logistic regression model was fitted using long-term ABU status as a binary outcome and a logit link function. The factors ‘housing type’ and ‘BES category’ are per definition constructed from other covariates and were therefore excluded from the model. Because the remaining variables considered to predict ABU and long-term ABU are numerous and expected to be correlated due to their association with increasing frailty, a stepwise selection procedure was used to identify the most significant explanatory variables. *P* values of < 0.05 were considered to indicate significance.

## Results

### Study population

Of the 146 residents, 116 were eligible and screened for ABU at baseline (S1a). Thirty residents were not enrolled because no informed consent was received (*n* = 23), because they did not meet the inclusion criteria (n = 2), deceased before sampling (*n* = 3) or were unable to deliver a urine sample (n = 2). Of the 116 that participated, 101 and 97 participants were included in the analyses of sampling round S1 and S2, respectively. Fifteen (S1) and 19 (S2) participants were excluded because of catheterization, antibiotic treatment, suspected urinary tract infections, not being able to give a urine sample, withdrawing from the study or being deceased. Six participants were included only in the analysis of S2, but not of S1. Ninety-one participants were included in both analyses.

The median age of the participants included in the analysis was 86 (range 67–104) at baseline for both S1 and S2, with participants housed in the residential home being only slightly younger (median age 84, range 71–93) than those accommodated in the nursing home (median age 86, range 67–104). Most of the participants were female (80%). Dementia (S1: 23%, S2: 22%), continuous incontinence (S1: 40%, S2: 36%) and being wheelchair-enabled (S1: 37%, S2: 32%) were common conditions. Detailed characteristics of the study population are described in Table 1. Importantly, none of the residents received prophylactic antibiotics to prevent UTIs.

**Table 1:** Demographic and clinical characteristics of the participants and prevalence rates of asymptomatic bacteriuria in each subgroup at sampling rounds S1 and S2

	S1		S2	
	frequency n (%)	ABU prevalence n (%)	frequency n (%)	ABU prevalence n (%)
Overall	101 (100.0)	40 (39.6)	97 (100.0)	40 (41.2)
Age				
Age > 85	52 (51.5)	24 (46.2)	51 (52.6)	26 (51.0)
Age ≤ 85	49 (48.5)	16 (32.7)	46 (47.4)	14 (30.4)
Gender				
Female	81 (80.2)	37 (45.7)	78 (80.4)	37 (47.4)
Male	20 (19.8)	3 (15.0)	19 (19.6)	3 (15.8)
LTCF unit				
Nursing home	78 (77.2)	37 (47.4)	70 (72.2)	37 (52.9)
Residential home	23 (22.8)	3 (13.0)	27 (27.8)	3 (11.1)
BES category				
Cd	25 (24.8)	19 (76.0)	21 (21.6)	18 (85.7)
A,B,C	44 (43.6)	18 (40.9)	41 (42.3)	18 (43.9)
O	32 (31.7)	3 (9.4)	35 (36.1)	4 (11.4)
Mobility				
Wheelchair-enabled	37 (36.6)	17 (45.9)	31 (32.0)	19 (61.3)
Ambulatory	63 (62.4)	23 (36.5)	65 (67.0)	21 (32.3)
Bedridden	1 (1.0)	0 (0.0)	1 (1.0)	0 (0.0)
Comorbidities				
Dementia	23 (22.8)	17 (73.9)	21 (21.6)	16 (76.2)
No dementia	78 (77.2)	23 (29.5)	76 (78.4)	24 (31.6)
Diabetes	23 (22.8)	11 (47.8)	20 (20.6)	11 (55.0)
No diabetes	78 (77.2)	29 (37.2)	77 (79.4)	29 (37.7)
Continuous incontinence	40 (39.6)	27 (67.5)	35 (36.1)	27 (77.1)
Occasional incontinence	30 (29.7)	8 (26.7)	30 (30.9)	9 (30.0)
No incontinence	31 (30.7)	5 (16.1)	32 (33.0)	4 (12.5)
Treatments				
Diuretics treatment	41 (40.6)	16 (39.0)	42 (43.3)	15 (35.7)
No diuretics treatment	60 (59.4)	24 (40.0)	55 (56.7)	25 (45.5)
Analgesics treatment	47 (46.5)	18 (38.3)	43 (44.3)	17 (39.5)
No analgesics treatment	54 (53.5)	22 (40.7)	54 (55.7)	23 (42.6)

### Prevalence rates of ABU

**Fig. 2:**
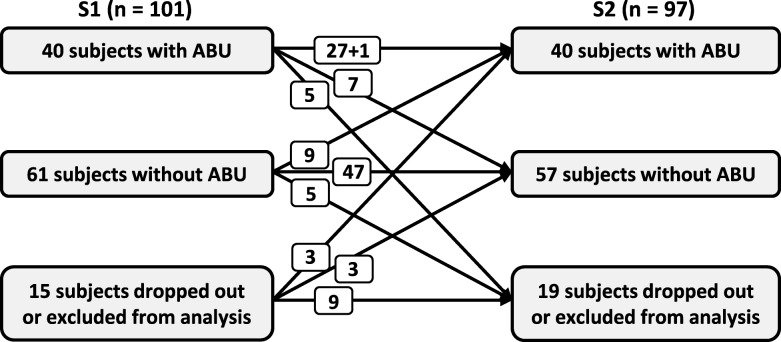
Gain and loss of asymptomatic bacteriuria during the 3-month study period

In each sampling round, ABU occurred in 40 participants (S1: 40%; S2: 41%). During the 3-month study period, 7 out of 40 subjects with ABU in S1 no longer had ABU in S2, while 9 acquired ABU newly by the time of S2 (Fig. 2). In one participant, the species causing ABU changed from *Escherichia coli* in S1 to *Citrobacter koseri* in S2. These 17 cases were defined as transient ABU. Twenty-seven of the 91 participants (30%) that participated in both sampling rounds had ABU with the same species throughout S1 and S2 and were defined as long-term ABU.

### Etiology

All 305 urine cultures included in the analysis showed growth of bacteria. Positive urine cultures (> 10^5^ CFU/ml) were obtained in 188 (62%) cases, while all remaining cultures were considered contaminated.

Fourteen species were identified as causative of ABU (see Additional file 1). *E. coli* was the most common cause and occurred in 35/40 (88%; S1) and 32/40 (80%; S2) of the subjects with ABU. ABU with *Aerococcus urinae* was identified in one case in S1 (3%) and three cases in S2 (7%). All other species caused no more than one ABU case (2.7%) per sampling round. When distinguishing by gender, *E. coli* occurred in 92% (S1) and 84% (S2) of all women with ABU (see Additional file 1). In men, *E. coli* caused one out of the three ABU cases in each sampling round. However, the small total number of ABU cases in men does not allow for interpretation.

Of the 91 participants who were included in both S1 and S2, 28 (31%) had ABU in both sampling rounds. Of those, 27/91 (30%) had positive urine cultures with the same species at all four sampling points, indicating that they carried the same organism continuously for at least 3 months. In women, these cases of long-term ABU were mostly caused by *E. coli* (24/26, 92%), while *A. urinae* and *Klebsiella variicola* caused one case each. In men, the only case of long-term ABU was caused by *Streptococcus agalactiae.*

Out of the 188 positive urine cultures, 34 (18%) showed growth in concentrations of > 10^5^ CFU/ml with more than one species. In five cases, two consecutive samples yielded positive urine cultures with the same two or three species, indicating co-colonization. These cases of polymicrobial ABU were caused by (1) *E. coli + A. urinae*, (2) *E. coli + Staphylococcus haemolyticus*, (3) *E. coli + Lactobacillus delbrueckii*, (4) *E. coli + Klebsiella oxytoca + Streptococcus gallolyticus* and (5) *E. coli + Aerococcus sanguinicola + A. urinae*.

### Risk factors for ABU

**Fig. 3:**
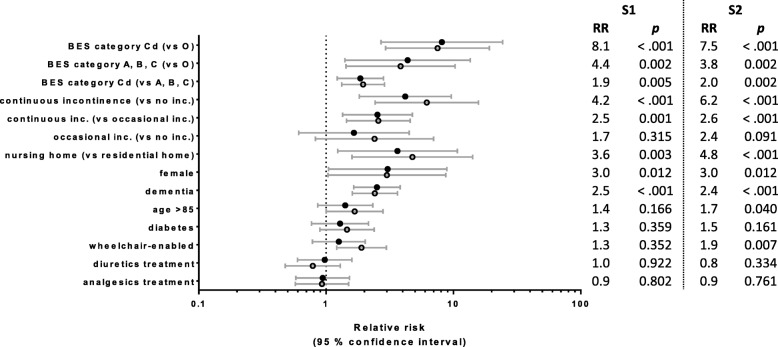
Relative risks (RR) and significance levels (*p*) for asymptomatic bacteriuria associated with potential risk factors in sampling rounds S1 (closed circle) and S2 (open circle). Horizontal lines indicate 95% confidence intervals

Prevalence rates of ABU associated with demographic and clinical characteristics of the participants are shown in Table 1. Figure 3 shows the relative risk of having ABU associated with potential risk factors. The strongest predictor for ABU was the Belgian evaluation scale (BES), which describes the dependency of older adults in activities of daily living. Prevalence rates of ABU increased from 9 to 11% in the group of independent participants (category O) to 41–44% in participants with intermediate dependencies (category A, B and C) and 76–86% in the group of highly dependent and disoriented participants (category Cd), corresponding to a 7.5–8.1fold increased relative risk for participants in category Cd when compared to participants in category O. Similarly, the prevalence of ABU among residents of the nursing home (47–53%) was significantly higher when compared to residents of the residential home (11–13%).

Participants with continuous urinary incontinence were 4.2 (S1) to 6.2 (S2) times more likely to have ABU than participants without incontinence, while participants with occasional urinary incontinence had an intermediate relative risk (RR = 1.7–2.4). The prevalence of ABU was significantly higher in women than in men (46–47% vs 15–16%, RR = 3). Dementia was also strongly associated with the risk of having ABU (RR = 2.4–2.5). Diabetes, using a wheelchair, and higher age (> 85 years) increased the risk of having ABU, but these associations were not significant or reached significance only in one of the two sampling rounds. Diuretics and analgesics use was documented because of a potential impact on bacterial clearance by diuresis and on the ability of patients to recognize symptoms of a UTI, respectively. However, receiving analgesics or diuretics did not influence the prevalence of ABU.

Despite gain and loss of ABU during the two sampling rounds and partially different participants included in the analyses of S1 and S2, the prevalence rates and resulting relative risks differed only slightly between S1 and S2, with the exception of the associations of wheelchair use and increased age with ABU, which were significant only in S2.

To account for a potential correlation between risk factors, the relationship between ABU and a subset of these risk factors was additionally assessed using a multivariate logistic regression model. The factors that were strongly associated with ABU (*p* < 0.2) in the univariate analyses of either S1 or S2, i.e. continuous incontinence, occasional incontinence, gender, dementia, diabetes, mobility, and age, were considered for inclusion in the final model. BES category and housing type both depend, by definition, on other risk factors and were excluded from the model. This multivariate analysis revealed that risk factors significantly associated with ABU were female gender (OR = 6.3) and continuous incontinence (OR = 15.9 vs no incontinence, OR = 8.5 vs occasional incontinence), while dementia, diabetes, mobility, and age were found to be correlated with those (Table 2).

**Table 2:** Odds ratios (OR) and 95% confidence intervals (CI) for parameters in logistic regression models

	OR	95% CI
Female gender (vs male)	6.3*	1.8–22.5
Continuous incontinence (vs no)	15.9*	5.8–43.5
Continuous incontinence (vs occasional)	8.5*	3.2–23.3
Occasional incontinence (vs no)	1.9	0.6–5.4

Parameters were applied to sampling rounds S1 and S2 combined while acknowledging for the association between observations from the same patient through GEE. Asterisks indicate statistical significance (*p* < 0.05).

Combined effects of the strongest predictors of ABU as identified in the univariate and multivariate analysis resulted in prevalence rates of 79–94% among female participants suffering from continuous incontinence or being assigned to BES category Cd (Table 3).

**Table 3:** Combined effects of risk factors: prevalence rates of asymptomatic bacteriuria in female participants exposed to the main predictors for ABU

	ABU in S1 n (%)	ABU in S2 n (%)
Female participants with continuous incontinence	26/33 (79%)	24/29 (83%)
Female participants in BES category Cd	18/21 (86%)	14/15 (94%)

### Risk factors for transient and long-term ABU

Risk factors associated with long-term ABU (ABU episodes in S1 and S2) and transient ABU (ABU episode in S1 or S2) were evaluated.

**Fig. 4:**
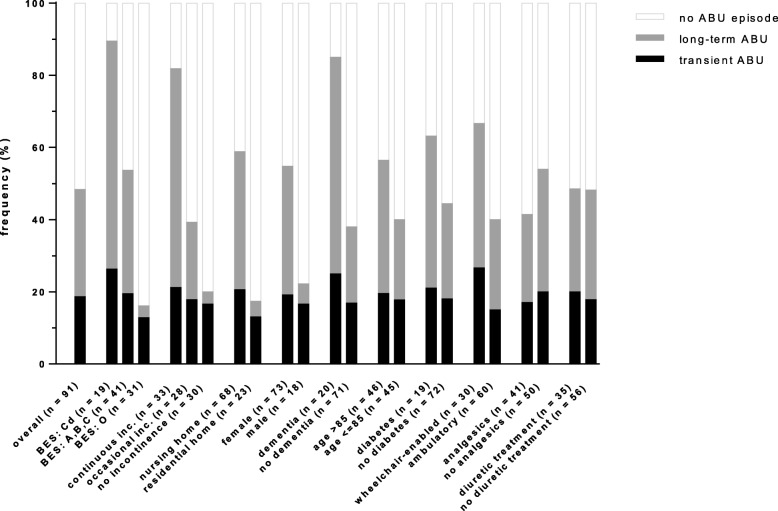
Frequency of long-term and transient asymptomatic bacteriuria in the subpopulation of 91 subjects who participated in both sampling rounds

Transient ABU occurred in 17 out of the 91 participants (19%) who were included in both sampling rounds. Prevalence variations fell within a relatively narrow range across different risk factor associated subgroups, with prevalence rates ranging from 13% in residential home residents and independent subjects (BES category O) to 27% in subjects using a wheelchair (Fig. 4 and Additional file 2). Although prevalence rates were higher in the groups exposed to the main ABU risk factors when compared to the respective non-exposed groups, the differences were not statistically significant. This result is partially attributable to the small number of participants with transient ABU.

Long-term ABU occurred in 27 out of 91 participants (30%). In contrast to transient ABU, the prevalence rates of long-term ABU were much more variable and could be as low as 3% in groups of participants not exposed to risk factors, while reaching up to 63% in certain risk-factor exposed groups. Participants with continuous urinary incontinence, dementia and of female gender were 18.2 (*p* < 0.001), 2.8 (*p =* 0.002) and 6.4 (*p =* 0.012) times as likely to develop long-term ABU when compared to continent, non-dementing and male participants, respectively. This was again reflected by high relative risk values for BES categories, with the most dependent (category Cd) and intermediately dependent participants (category A,B,C) having 19.6 (*p* < 0.001) and 10.6 (*p =* 0.001) times the risk of long-term ABU compared to the most independent participants (category O). Correspondingly, the prevalence of long-term ABU in the nursing home was 8.8 times (*p =* 0.002) higher than in the residential home.

In addition to the univariate analysis, a multivariate analysis was used to define independent predictors of long-term ABU. BES category and housing type were excluded as variables. Here, only female gender and continuous incontinence were significantly associated with long-term ABU (Table 4), while the other variables correlated with these two or did not significantly contribute to the multivariate model.

**Table 4:** Odds ratios (OR) and 95% confidence intervals (CI) for parameters in the logistic regression model for long-term ABU

	OR	95% CI
Female gender (vs male)	10.3*	1.1–96.6
Continuous incontinence (vs no)	43.5*	5.1–333.3
Continuous incontinence (vs occasional)	7.0*	2.1–23.3
Occasional incontinence (vs no)	6.2	0.7–55.6

Asterisks indicate statistical significance (*p* < 0.05)

### Evaluation of UTI episodes during a 6-month follow up period

To assess a potential role of ABU in preventing UTI, participants were evaluated for the occurrence of UTI episodes during the study and the 6 months after the study. Only six UTI cases which fulfilled the McGeer surveillance criteria were recorded. Of those who had long-term ABU (n = 27), none were treated for UTI. Two participants who had ABU in either S1 or S2, while being negative or excluded in the other sampling round (*n* = 25), were treated for UTIs, both caused by *E. coli*, the same species as was found during the ABU episode. One of those presented with unspecific symptoms only, while the other reported a costovertebral angle pain. Four UTI cases occurred among those with no prior record of ABU (*n* = 55). Causative species were *E. coli* (twice), *Enterobacter cloacae* and *Proteus mirabilis*, and all of those presented with typical UTI symptoms (at least one of the following: dysuria, urgency, increased frequency, suprapubic pain).

## Discussion

In the general population, ABU is uncommon, and long-term ABU is rare, occurring in less than 5% and fewer than 1% of healthy women, respectively [20]. In LTCF residents, the prevalence of ABU is much higher and is estimated to occur in 25 to 50% of women [2, 13]. In our study, we demonstrate that on average 40% of the LTCF residents present with ABU, in almost half of all women and about 15% of men. During the 3-month period, 18% of initially positive participants lost ABU, while 15% of initially negative participants gained ABU, indicative of its transient nature and in line with the high turnover rates and high rate of recolonization reported by Rodhe et al. [21] in a cohort of non-institutionalized residents of over 80 years. Thirty percent of our participants, 36% of all female subjects and one male (6%) subject, were shown to have long-term ABU, i.e. over the whole 3-month study period. Despite the different timeframes used in the few longitudinal studies described, such persistent ABU was reported to occur in up to 25% of the population of older adults [11, 15, 22]. These data confirm a high susceptibility to colonization among older adults, coupled with decreased spontaneous resolution when compared to other populations [23].

The etiology of ABU in older adults can be diverse, but *Escherichia coli* is by far the most common species with reported peak prevalence rates of 80 and 60% for community-dwelling and institutionalized older adults, respectively [24]. The most common causal species of ABU in our study was *E. coli*, which was found in about 84% of all participants and 90% of all women with ABU. Second most common was *A. urinae* (5%), which is occasionally reported as a uropathogen and, because it is more difficult to culture and to identify, might have been missed in earlier studies [25]. Other *Enterobacteriaceae*, non-fermentative Gram-negative bacilli, and Gram-positive bacteria were also shown to be causative of ABU [24], which is in line with other species recovered in this study. In clinical routine, urine cultures with three or more species are usually considered to be contaminated and a new sample is requested. In this study, urine cultures with multiple species were not per se excluded, as a confirmative second sample was requested from all participants with a positive initial urine culture. A few cases of potential polymicrobial ABU were found, always consisting of *E. coli* together with one or two other species, all above the 10^5^ CFU/ml threshold in two consecutive samples. As these cases of polymicrobial ABU comprised species including *S. haemolyticus*, *S. epidermidis,* and *L. delbrueckii*, known to be part of the typical skin and periurethral flora [26], they might be indicative of contamination, despite the two consecutive samplings and the high bacterial counts. Alternatively, as such Gram-positive organisms have been reported to cause UTI and polymicrobial infections were shown to occur in the older adults [26], these might be true cases of polymicrobial ABU.

To assess the risk factors leading to ABU, clinical data were obtained from patient records and by using a questionnaire. Female gender, suffering from continuous urinary incontinence and dementia were all shown to be strongly associated with having ABU. Being diabetic and the use of a wheelchair or higher age (> 85 years) resulted in non-significant associations or reached statistical significance only in one of the two sampling rounds.

Incontinence has repeatedly been associated with ABU [12, 27], as is the case in our study, with incontinent participants having a 4–6 times higher chance of having ABU compared to continent participants. For physiological and anatomical reasons, women are more prone to ABU than men [28]. Urinary incontinence is more common among female than male older adults [29] and certainly additionally contributes to the increased risk for women, which, in this study, was found to be 3 times higher than for men. Although participants with dementia had a 2.5 times higher risk of having ABU, a multivariate analysis revealed that dementia was not independently associated with ABU. Dementia strongly correlated with incontinence, with roughly 90% of dementing older adults in our study suffering from continuous incontinence and the remaining 10% from occasional incontinence (data not shown), an observation which is in line with results by Skelly & Flint [30]. Such a lack of independent association of ABU with dementia or reduced mental capacity in older adults was similarly reported by Eberle et al. [12] and Rodhe et al. [27]. Nevertheless, dementia might serve as a valuable indicator for an increased risk of both incontinence and ABU.

Reduced mobility was earlier shown to result in a significantly higher chance of having ABU in older adults women [27], while our data are not conclusive. The first sampling round showed only a positive non-significant association, whereas in the second round we found an increased risk of ABU associated with using a wheelchair. Age is linked to several conditions which lead to ABU, explaining the high prevalence among the older adults [2]. However, among the very old, age is outweighed by other risk factors. In our population, being > 85 years of age was positively, but not significantly correlated with ABU. Other studies came to similar conclusions [15].

The Belgian Evaluation Scale (BES), an adaption of the Activities of Daily Living (ADL) index with additional score categories, classifies older adults based on their need for care [18]. As characteristics such as mobility, dementia, and incontinence are incorporated in the BES score, not surprisingly, this was shown to be the strongest predictor for ABU in our study, with the most dependent participants having a two times higher chance of having ABU (prevalence 76–86%) compared to somewhat dependent participants (prevalence 41–44%) and even an 8 times higher risk compared to the most independent group (prevalence 9–11%). This was also reflected in the housing type, the more dependent people living in the nursing home presenting with a 4–5 times higher risk of ABU and prevalence rates of 47–53% compared to rather independent subjects that are housed in the residential home with prevalence rates as low as 11–13%. As the residential home can be considered comparable to a community setting, these figures correspond well with what was reviewed by Nicolle [2], who found ABU prevalence rates of 15–50% in institutionalized versus 1.5–17% in non-institutionalized older adults.

The prevalence and risk factors associated with long-term ABU and transient ABU were also evaluated. The prevalence rates of transient ABU were between 13 and 27% for all analyzed risk factor groups, which is comparable to prevalence rates of general ABU in the community setting [2]. In contrast, prevalence rates of long-term ABU (> 3 months), causing a majority (61%) of all ABU cases in our study, were as low as 3% in continent or in independent participants (BES category O), while reaching peak prevalence rates of > 60% in the subgroups of participants with dementia, continuous incontinence or BES category Cd. Remarkably, in our study, the prevalence rates of ABU and long-term ABU in the subgroup of female participants suffering from continuous incontinence were 79–83 and 68%, respectively. Our results thus indicate a strongly increased chance of having ABU with increasing frailty, often leading to persistent colonization in the most fragile women.

These observations should be considered when evaluating screening, diagnosis and treatment options for suspected UTIs. On the one hand, antibiotic treatment of ABU in institutionalized older adults was shown not being beneficial and leading to adverse outcomes and antimicrobial resistance [7, 8]. On the other hand, it is assumed that ABU might even be protective of UTI by competing with pathogenic organisms and preventing more virulent strains to establish in the urinary tract [10]. In our study, we recorded the occurrences of UTIs during the study and in the 6 months after the two collections rounds. Although these data were not powered for subgroup analysis, we observed that LTCF residents with previous long-term ABU were less likely to experience a UTI than residents who had transient ABU or no ABU episode in the two collections rounds, hinting towards a protective effect of ABU.

While the identification of risk factors helps clinicians to better assess the potential need for treatment, there still is an urgent need for improved diagnostic tests differentiating ABU and UTI, especially in the geriatric population. This challenge could be overcome by improving the diagnosis of the inflammatory state of the urinary tract mucosa. Analysis of inflammation marker cytokine levels in urine was shown to be promising to distinguish ABU from upper urinary tract infections [31, 32]. Alternatively, the risk for a severe infection and the need for treatment could be predicted based on bacterial determinants. Several virulence factors in *E. coli* were earlier shown to be associated with pyelonephritis [33]. Given the recent advances in molecular diagnostics [34], a rapid molecular diagnostic test detecting the presence of *E. coli* and its pathogenicity potential could help to decide whether to give antibiotics or not. After such treatment, the administration of avirulent ABU strains in a probiotic-like manner might reduce the risk of reinfection with the virulent strain and might prove a superior strategy compared to merely giving antibiotics alone.

## Conclusion

In LTCFs, antimicrobial use for UTI is high and often the result of a misdiagnosis of ABU as UTI [1, 35]. Differentiating between ABU and UTI in this population is challenging. Identifying subpopulations with low or high risks for asymptomatic bacteriuria could help to guide the decision to initiate or withhold antibiotic therapy. In this study, we showed that female participants with urinary incontinence or assigned to BES group Cd had ABU prevalence rates of 80 to 90%. Vague UTI symptoms and positive urine cultures in these populations should be interpreted with care to avoid adverse effects of antibiotic treatment. In contrast, ABU prevalence rates were relatively low (10 to 15%) among institutionalized older adults without incontinence, of male gender or categorized into BES group O. In these subgroups, vague signs of a UTI combined with positive urine cultures are more likely to predict a true infection. Our study thus demonstrates that due to the high prevalence of ABU, the frailest population is at the highest risk of unnecessary treatment. Such knowledge should be more actively used when considering treatment strategies for the older adults. In the future, molecular diagnostics predicting bacterial pathogenicity or host susceptibility could help to identify those persons at risk of infection and requiring treatment.

## Additional files

Additional file 1: Causative species of asymptomatic bacteriuria identified in sampling rounds S1 or S2 separated by gender. (PDF 143 kb)

Additional file 2: Prevalence of long-term ABU and transient ABU in different risk factor groups. Long-term ABU and transient ABU cases among 91 participants who were included in the analysis of both sampling rounds S1 and S2 (no ABU episode: *n* = 47, transient ABU: *n* = 17, long-term ABU: *n* = 27). (PDF 112 kb)

## Data Availability

The datasets used and/or analyzed during the current study are available from the corresponding author on reasonable request.

## Abbreviations

ABU: Asymptomatic bacteriuria
ADL: Activities of Daily Living
BES: Belgian Evaluation Scale
CI: Confidence intervals
LTCF: Long-term care facility
OR: Odds ratio
RR: Relative risk
UTI: Urinary tract infection
