# Supplementary material for: Asymptomatic bacteriuria in older adults: the most fragile women are prone to long-term colonization
Source: BMC Geriatr. 2019 Jun 21;19:170. doi: 10.1186/s12877-019-1181-4 (PMC6588879; doi:10.1186/s12877-019-1181-4)
Supplement: Supplementary file 1 — Causative species of asymptomatic bacteriuria identified in sampling rounds S1 or S2 separated by gender. (PDF 143 kb) [file 12877_2019_1181_MOESM1_ESM.pdf]

Additional file 1. Causative species of asymptomatic bacteriuria.

species	women		men	
	**S1 (n = 37) n (%)***	**S2 (n = 37) n (%)***	**S1 (n = 3) n (%)**	**S2 (n = 3) n (%)**
E. coli	34 (91.9)	31 (83.8)	1 (33.3)	1 (33.3)
A. urinae	1 (2.7)	3 (8.1)	0 (0)	0 (0)
K. pneumoniae	1 (2.7)	1 (2.7)	0 (0)	0 (0)
K. variicola	1 (2.7)	1 (2.7)	0 (0)	0 (0)
K. oxytoca	1 (2.7)	0 (0)	0 (0)	0 (0)
S. haemolyticus	1 (2.7)	0 (0)	0 (0)	0 (0)
S. gallolyticus	1 (2.7)	0 (0)	0 (0)	0 (0)
E. faecalis	0 (0)	1 (2.7)	1 (33.3)	0 (0)
C. koseri	0 (0)	1 (2.7)	0 (0)	0 (0)
M. morganii	0 (0)	1 (2.7)	0 (0)	0 (0)
L. delbrueckii	0 (0)	1 (2.7)	0 (0)	0 (0)
A. sanguinicola	0 (0)	1 (2.7)	0 (0)	0 (0)
S. epidermidis	0 (0)	0 (0)	0 (0)	1 (33.3)
S. agalactiae	0 (0)	0 (0)	1 (33.3)	1 (33.3)

Causative species of asymptomatic bacteriuria identified in sampling rounds S1 or S2, separated by gender.
*total exceeds 100% as polymicrobial ABU occurred in three cases
