# Supplementary material for: Asymptomatic bacteriuria in older adults: the most fragile women are prone to long-term colonization
Source: BMC Geriatr. 2019 Jun 21;19:170. doi: 10.1186/s12877-019-1181-4 (PMC6588879; doi:10.1186/s12877-019-1181-4)
Supplement: Supplementary file 2 — Prevalence of long-term ABU and transient ABU in different risk factor groups. Long-term ABU and transient ABU cases among 91 participants who were included in the analysis of both sampling rounds S1 and S2 (no ABU episode: n = 47, transient ABU: n = 17, long-term ABU: n = 27). (PDF 112 kb) [file 12877_2019_1181_MOESM2_ESM.pdf]

Additional file 2. Prevalence of long-term ABU and transient ABU in different risk factor groups.

	prevalence (%)			
	long-term ABU		transient ABU	
	exposed	non-exposed	exposed	non-exposed
**age >85**	37.0	22.2	19.6	17.8
**female gender**	35.6	5.6	19.2	16.7
**nursing home (vs residential home)**	38.2	4.3	20.6	13.0
**BES category Cd (vs O)**	63.2	3.2	26.3	12.9
**BES category Cd (vs A, B, C)**	63.2	34.1	26.3	19.5
**BES category A, B, C (vs O)**	34.1	3.2	19.5	12.9
**wheelchair-enabled**	40.0	25.0	26.7	15.0
**dementia**	60.0	21.1	25.0	16.9
**diabetes**	42.1	26.4	21.1	18.1
**continuous incontinence (vs no inc.)**	60.6	3.3	21.2	16.7
**incontinence (vs occasional inc.)**	60.6	21.4	21.2	17.9
**occasional incontinence (vs no inc.)**	21.4	3.3	17.9	16.7
**diuretics treatment**	28.6	30.4	20.0	17.9
**analgesics treatment**	24.4	34.0	17.1	20.0

Long-term ABU and transient ABU cases among 91 participants who were included in the analysis of both sampling rounds S1 and S2 (no ABU episode: n = 47, transient ABU: n = 17, long-term ABU: n = 27).
